# Single-cell RNA sequencing reveals pancreatic cancer microenvironment heterogeneity for precision therapy

**DOI:** 10.1515/jtim-2026-0013

**Published:** 2026-02-18

**Authors:** Yaya Bai, Shimin Wang, Chunhua Zhou, Duowu Zou

**Affiliations:** Department of Gastroenterology, Ruijin Hospital, Shanghai Jiao Tong University School of Medicine, Shanghai, China

Pancreatic cancer is one of the most malignant tumors of the digestive system. Currently, its global incidence is gradually increasing, with mortality rates closely aligning with incidence rates. Its median survival time is 4 months, and the 5-year survival rate is only 13%.^[1,2]^ Despite ongoing research progress in the field of pancreatic cancer, its survival rate remains the lowest among all malignant tumors. The significant heterogeneity and complexity inherent in both pancreatic tumor cells and the tumor microenvironment (TME) render the management of pancreatic cancer particularly challenging. Consequently, a deeper understanding of the TME is crucial for advancing precision therapy in this disease.

In recent years, the emergence of single-cell RNA sequencing (scRNA-seq) technology,^[3]^ like a high-precision surgical scalpel, has enabled researchers to dissect the complex ecosystem of pancreatic cancer gradually. Traditional high-throughput sequencing technologies in the study of pancreatic cancer primarily focused on identifying “driver genes” associated with tumor initiation and progression, yet overlooked tumor heterogeneity. As an emerging technology, scRNA-seq overcomes key limitations of conventional sequencing by enabling a deep, multi-omic exploration of malignant tumors at single-cell resolution. This provides insights into tumor heterogeneity, tumorigenesis, progression, drug resistance, and therapeutic strategies.

Through scRNA-seq, researchers have uncovered the diversity of cell types within pancreatic tumor tissues, comprising both cancer cells and the various stromal and immune cells of the TME.^[4]^ These cell types play distinct roles in the tumorigenesis, progression, and treatment of pancreatic cancer, making them a focal point of research interest. One study investigated the origin and function of transcriptionally diverse cancer-associated fibroblast (CAF) populations in pancreatic ductal adenocarcinoma (PDAC).^[5]^ Using scRNA-seq, the researchers revealed that tumor-intrinsic SETD2 deficiency releases BMP2 signaling through the ectopic acquisition of H3K27Ac, leading to the differentiation of CAFs into a lipid-rich phenotype. These lipid-rich CAFs then supply lipids for mitochondrial oxidative phosphorylation *via* the ABCA8a transporter, thereby enhancing tumor progression. This study not only links CAF heterogeneity to epigenetic dysregulation in tumor cells but also uncovers a previously underappreciated metabolic interaction between CAFs and pancreatic tumor cells. Furthermore, the research proposes targeting oxidative phosphorylation as a potential therapeutic strategy for PDAC patients with SETD2 deficiency, offering new perspectives for precision treatment.

Additionally, researchers have utilized scRNA-seq to elucidate the effects of chemotherapy on pancreatic cancer patients and their TME. In one study, researchers collected fresh tissue samples from PDAC patients before and after chemotherapy and performed scRNA-seq to analyze the transcriptomic profiles of PDAC.^[6]^ This approach revealed heterogeneity in the composition of epithelial malignant subtypes and examined the expression of inhibitory checkpoint molecules and ligand-receptor interactions before and after chemotherapy. The results demonstrated that transcriptional differences among patients primarily resided in the TME. Following chemotherapy, alterations were observed in the TME, where reduced TIGIT receptor expression on CD8^+^ T cells contributed to tolerance in immunotherapy. Another study used single-cell spatial transcriptomics to dissect the remodeling of multicellular neighborhoods and cell-cell interactions in human pancreatic cancer following neoadjuvant chemotherapy and radiotherapy.^[7]^ This research identified functional enrichment in interleukin-6 family signaling that confers chemotherapy resistance. In our team’s latest study, scRNA-seq was performed on matched pre- and post-treatment tumor biopsies and peripheral blood mononuclear cells from 28 PDAC patients treated with abraxane plus gemcitabine. This approach aimed to uncover how chemotherapy remodels tumor cell plasticity and the TME, thereby influencing clinical outcomes. Our integrative analysis uncovered a chemo-resistant niche, comprising SNCG^+^ basal-like tumor cells, SPP1^+^ tumor-associated macrophages, and exhausted T cells, which progressively dominated the TME during treatment in non-responders. This niche was driven by the dual-function immune checkpoint CD276/B7-H3, which facilitates the acquisition of a chemo-resistant basal-like state in tumor cells, while simultaneously inducing T cell exhaustion and enhancing the angiogenic capacity of macrophages. By elucidating the remodeling of PDAC tumor cell plasticity and its interplay with the TME during chemotherapy, our work thereby establishes CD276/B7-H3 as a critical regulator and a viable therapeutic target to counter chemoresistance.

Furthermore, by revealing key insights, scRNA-seq is playing a crucial role in paving new paths for immunotherapy in pancreatic cancer. One study utilized scRNA-seq data to predict tumor-reactive T-cell receptors (TCR) for personalized T-cell therapy.^[8]^ By integrating high-throughput TCR clonality and reactivity validation techniques, the researchers developed a machine learning classifier named predicTCR. This tool leverages single-cell tumor-infiltrating lymphocyte (TIL) RNA sequencing data to accurately identify individual tumor-reactive TILs in an antigen recognition-independent manner. The technology enables prediction of tumor-reactive TCRs within days, thereby prioritizing TCR clonotypes and significantly accelerating the development of personalized T-cell therapies. Moreover, other researchers applied scRNA-seq to analyze the cellular landscape of Trp53-mutant mouse models driven by KrasG12D or KrasG12V, with either wild-type or deleted Smad4.^[9]^ The findings indicated that SMAD4 loss differentially altered the TME in KrasG12V PDAC compared to KrasG 12D PDAC, with the malignant compartment lacking JAK/STAT signaling dependency. Thus, the genotype of malignant cells influences the phenotypes of both tumor cells and stromal cells in PDAC, directly affecting therapeutic efficacy. In our previous work, we innovatively integrated multi-omics data to elucidate the role of pancreatic stellate cells (PSCs) in pancreatic cancer initiation and progression. We systematically compared the characteristics of PSCs in adjacent non-tumor tissues and tumor tissues, uncovering for the first time the key regulatory mechanisms underlying the quiescent-to-activated transition and identifying four functionally distinct tumor-associated PSC (TPSC) subpopulations.^[10]^ The study revealed that stress and hypoxia signals drive transcriptional and epigenetic remodeling of PSCs, leading to their activation and differentiation into the following subtypes: (1) CCL19^+^ TPSCs, enriched in immune cell-dense regions; (2) MYH11^+^ TPSCs, localized predominantly in nonmalignant stromal areas; (3) PLXDC1^+^ TPSCs, which possess the potential to differentiate into cancer-associated myofibroblasts (myCAFs). These PLXDC1^+^ TPSCs are distributed in stromal regions at the border of malignant tumor cells and are significantly associated with poor prognosis. For the first time, we systematically revealed the heterogeneity of PSCs in pancreatic cancer at single-cell and spatial resolution, challenging the conventional homogeneous view of activated PSCs. This provides a new perspective for TME research. We further uncovered a fibrotic-immunosuppressive microenvironment synergy mechanism: PLXDC1^+^ TPSCs were found to form a spatially co-localized network with LRRC15^+^ myCAFs and SPP 1^+^ macrophages at the tumor boundary. Through cellular interactions, this network drives CD8^+^ T cell exhaustion, representing the first spatial dissection of the stromal regulatory mechanism underlying immune evasion in pancreatic cancer.

**Figure 1:**
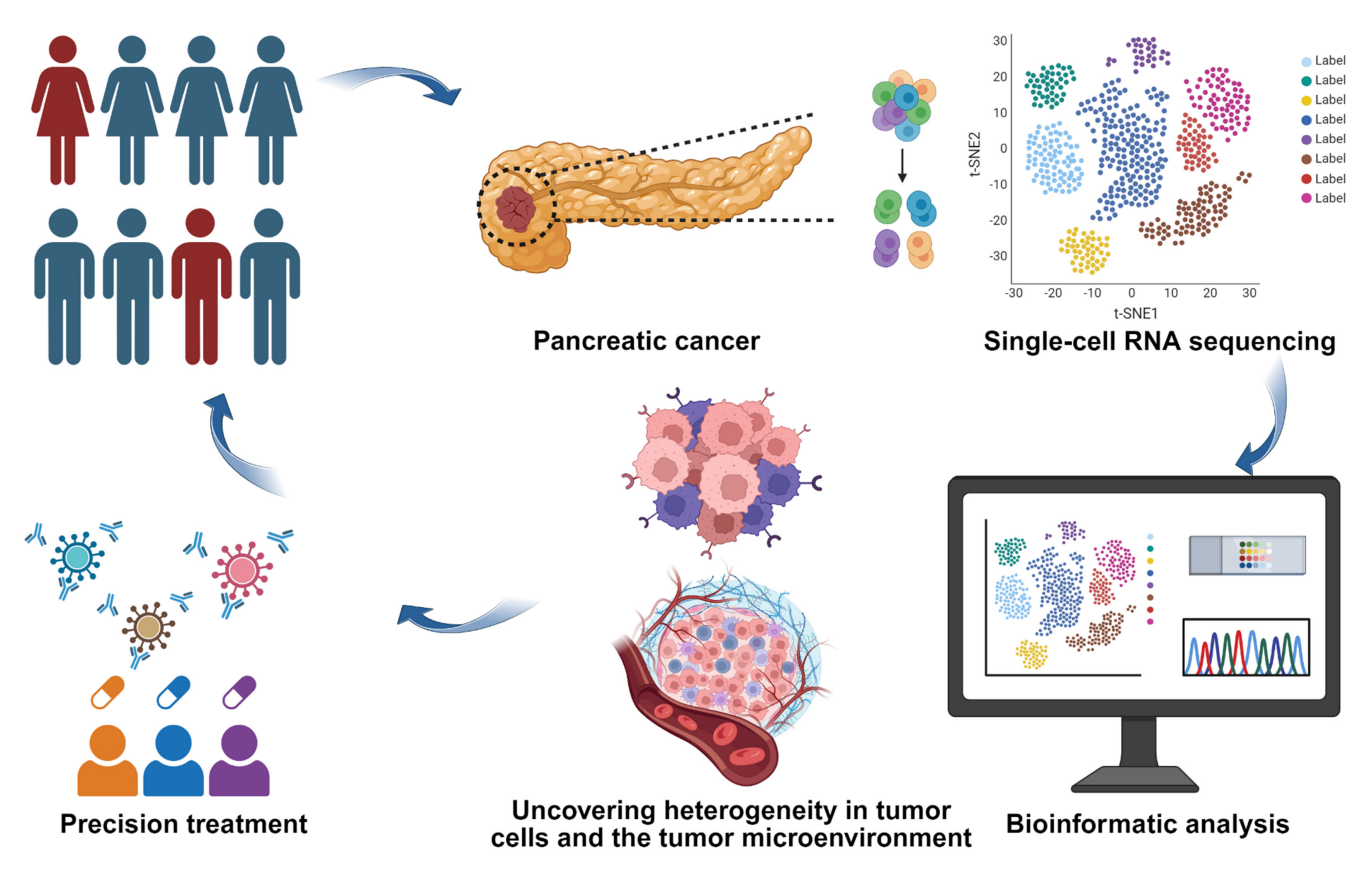
Precision medicine in pancreatic cancer based on single-cell RNA sequencing.

In summary, scRNA-seq is unveiling the true landscape of pancreatic cancer with unprecedented resolution (Figure 1). However, this approach faces challenges such as high technical noise, loss of information, and difficulties in capturing dynamic biological processes. For instance, the extremely low starting amount of nucleic acid in individual cells results in significant technical noise and a high rate of transcript dropout, particularly affecting low-abundance genes. Additionally, tissue dissociation erases the native spatial architecture of cells and their intercellular communication networks. Spatial transcriptomics^[11]^ enables *in situ* gene expression profiling while maintaining spatial information. But its application is limited by a fundamental trade-off between spatial resolution and analytical throughput, namely the number of detectable molecular targets. To overcome this limitation, the primary strategy currently involves integrating spatial transcriptomics with scRNA-seq through computational approaches.^[12]^

This integration leverages the high-resolution cell-type information from scRNA-seq to deconvolute each spot in spatial data, thereby inferring the composition and spatial distribution of different cell types within it. It is profoundly transforming research paradigms across multiple fields, particularly in deciphering the TME. Nonetheless, transcriptomics provides only gene activity and does not capture the functional states of proteins, which are the ultimate executors of cellular functions. Proteomics can validate gene predictions and uncover translational regulation and modifications, thereby providing a comprehensive understanding of the mechanisms underlying biological processes. Therefore, integrating scRNA-seq, spatial transcriptomics, and proteomics will bridge the limitations of individual technologies and enable the construction of a more holistic cellular atlas of the pancreatic cancer TME.^[13]^ This integration will drive research in pancreatic cancer from static sequencing toward a deeper understanding of functional dynamics. Meanwhile, by deciphering the spatiotemporal heterogeneity of tumors, microenvironmental interactions, and evolutionary trajectories, it will help clinical trials achieve precise enrollment, dynamic monitoring, and mechanismguided therapy, ultimately improving treatment success rates for pancreatic cancer. As deep learning further integrates with these technologies, researchers will not only be poised to transform pancreatic cancer from a “terminal disease” into a manageable condition but also establish new paradigms for treating other intractable malignancies. In the future, precision therapy will empower clinicians with more sophisticated tools to combat pancreatic cancer, often termed the “king of cancers”.
